# Evaluating Impacts of Different Omega-6 to Omega-3 Fatty Acid Ratios in Corn–Soybean Meal-Based Diet on Growth Performance, Nutrient Digestibility, Blood Profiles, Fecal Microbial, and Gas Emission in Growing Pigs

**DOI:** 10.3390/ani10010042

**Published:** 2019-12-24

**Authors:** Dinh Hai Nguyen, Hyeok Min Yun, In Ho Kim

**Affiliations:** Department of Animal Resource and Science, Dankook University, Cheonan-si, Chungnam 31116, Korea; nguyenhaibg90@gmail.com (D.H.N.); yunhm822@naver.com (H.M.Y.)

**Keywords:** blood profile, fecal gas emission, fecal microbial, nutrient digestibility, omega-6: omega-3 fatty acids, growth performance

## Abstract

**Simple Summary:**

There are two major classes of polyunsaturated fatty acids found in the diet, omega-3 and omega-6, these polyunsaturated fatty acids are essential because they cannot be synthesized by humans and animals. Also, they cannot be converted into each other in the body. Supplementation of omega-3 has been reported to improve the performance and health of pigs. However, during the past years, studies had demonstrated that animal performance could be influenced by the dietary level or the ratio of omega-6 and omega-3. A high omega-6: omega-3 ratio in diets promotes the pathogenesis of many diseases, including cardiovascular disease, cancer, and inflammatory and autoimmune diseases. An optimal omega-6: omega-3 ratio could inhibit immune stimulation to ensure the availability of more energy and nutrients for high performance and the homeostatic pathway. The present study investigated the effects of different omega-6: omega-3 ratios (17:1, 15:1, 10:1, 5:1) on growth performance, nutrient digestibility, blood profiles, fecal microflora, and gas emission in growing pigs. The results of this study showed that reducing omega-6: omega-3 ratio from 17:1 to 5:1 by increasing omega-3 in the diet increased body weight, energy digestibility, and reduced the low-density lipoprotein concentrations of blood in growing pigs.

**Abstract:**

This study was conducted to evaluate the effect of different omega-6: omega-3 fatty acid (FA) ratios in a corn–soybean meal-based diet in growing pigs. A total of 140 [Duroc × (Landrace × Yorkshire)] growing pigs with an average body weight (BW) of 24.75 ± 1.43 kg were used in a 6-week trial. Pigs were allocated randomly into one of four treatments according to sex and BW (seven replications with five pigs per pen). The treatment groups consisted of 4 diets with omega-6:omega-3 FA ratios of 17:1, 15:1, 10:1, and 5:1. In the current study, the energy digestibility, BW, and average daily gain (ADG) increased (*p* < 0.05) in pigs provided with the 5:1 diet compared to pigs fed the 17:1 diet in the sixth week. The low-density lipoprotein (LDL) concentrations of blood were lower (*p* < 0.05) in pigs fed the 5:1 diet compared to the 17:1 and 15:1 diet. However, the fecal microflora and fecal gas emissions were unaffected (*p* > 0.05) by the different omega-6: omega-3 FA ratios in diets. In conclusion, reducing omega-6: omega-3 ratio by increasing omega-3 in diet improved BW, ADG, and gross energy digestibility, and reduced the LDL concentrations of blood in growing pigs.

## 1. Introduction

There are two major classes of polyunsaturated fatty acids (PUFA) found in the diet, omega-3, and omega-6, which are essential but cannot be synthesized by humans and animals as well as cannot be transformed one to another in the body [1,2]. Hence, PUFA are crucial components of the food or diet. Beneficial effects of additions of essential fatty acids (FA) to diets fed to animals have been reported. Previous studies indicated that consuming omega-3 FA improved the health status of weanling pigs that are faced with different stressors due to their effects on the immune system [3,4]. Similarly, Upadhaya et al. indicated that providing omega-3 FA to pigs could improve performance and their health [5,6].

Studies have demonstrated that sow and cockerel performance could be influenced by the level or the ratio of omega-6 and omega-3 PUFA [7,8]. Simopoulos observed that in humans, the higher the ratio of omega-6: omega-3 FA in platelet phospholipids, the higher the death rate from cardiovascular disease. A high omega-6:omega-3 FA ratio in diets promotes the pathogenesis of many diseases, including cardiovascular disease, cancer, and inflammatory and autoimmune diseases [2]. Moreover, there exists a competition between the 18-carbon omega-3 and omega-6 FA because they share a common enzymatic pathway in the synthesis of their longer chain derivatives. Thus, the dietary ratio of omega-3 and omega-6 FA may be important to maximize the benefits of adding omega-3 FA into diets [9]. An optimal omega-6:omega-3 FA ratio could inhibit immune stimulation to provide more energy and nutrients for optimum performance and homeostatic pathways [10,11]. Previous reports showed that a diet with a lower omega-6:omega-3 PUFA ratio, rich in omega-3 PUFA, is beneficial for the growth performance and health of avian [12,13,14].

The costs of feed play a major role in determining the profitability of a pig producer because it represents up to 70% of the variable costs of swine production [15]. However, increasing the levels of omega-3 FA to reduce the ratio of omega-6: omega-3 FA may increase the cost of the feed. Thus, studies are focused to find techniques to enhance the effective delivery of omega-3 FA to appropriate sites in the body for absorption as well as reduce feed cost and extract more nutrients from feed ingredients. Nguyen et al. reported that the effectiveness of organic acids could be enhanced by matrix coating or encapsulation techniques to protect OAs for targeted delivery to different gut segments has gained considerable attention [16]. We hypothesize that using a coated omega-3 FA may also enhance the effective absorption, metabolism of omega-3 FA, and thus stimulate their modulatory effect on the large intestinal microbiota.

The object of the current study was to examine effects of different omega-6:omega-3 FA ratios (17:1, 15:1, 10:1, 5:1) in corn-soybean meal-based diets on growth performance, nutrient digestibility, blood profiles, fecal microflora, and gas emission in growing pigs. 

## 2. Materials and Methods

The experimental protocol used in this study was approved by the Animal Care and Use Committee of Dankook University.

### 2.1. Source of Omega-3 FA

The omega-3 FA used in this study was provided by a commercial company (Morningbio Co., Ltd., Cheonan, Korea). It was produced from linseed oil and refined fish oil and was protected using matrix coating technology. According to the information provided by the supplier, the omega-3 and omega-6 PUFA contents were 17.8% and 4.7% of dry matter, respectively.

### 2.2. Experimental Design, Animals, and Diets

A total of 140 [Duroc × (Landrace × Yorkshire)] growing pigs with an average body weight (BW) of 24.75 ± 1.43 kg were used in a 6-week trial. Pigs were allocated randomly into one of four treatments according to sex and BW, providing seven replicates with two gilts and three barrows per pen. The treatment groups consisted of four diets with omega-6:omega-3 FA ratios of 17:1, 15:1, 10:1, and 5:1. Diets were formulated to meet or exceed NRC recommendations for all nutrients (Table 1). The analyzed fatty acid profile of the experimental diets were shown in the Table 2. Pigs were housed in an environmentally controlled room with a slatted plastic floor [17]. Throughout the experiment, all pigs were provided with *ad libitum* access to feed and water through a self-feeder and nipple drinker, respectively. Target room temperature and humidity were 25 ± 1°C and around 60%, respectively.

### 2.3. Sampling and Measurements

Individual pigs’ BW and feed intake were measured initially and at the end of week 6 and feed consumption was recorded on a pen basis during the experiment to determine average daily gain (ADG), average daily feed intake (ADFI), and gain to feed ratio (G: F).

Chromium oxide was added to the diet as an indigestible marker at 0.20% of the diet for seven days prior to fecal collection at the end of week 6 for calculation of dry matter (DM), nitrogen (N), and gross energy digestibility. Fecal grab samples were collected at random from two pigs (one gilt and one barrow) from each pen and were mixed and pooled, and a representative sample was stored in a freezer at −20 °C until analysis. Fecal samples were dried at 70 °C for 72 h and finely ground to pass through a 1-mm screen. All feed and fecal samples were analyzed for DM (method 930.15) and crude protein (N 9 6.25; method 984.13) [18]. Gross energy was determined by measuring the heat of combustion in the samples, using a bomb calorimeter (Parr 6100; Parr Instrument Co., Moline, IL, USA). Chromium was analyzed via UV absorption spectrophotometry (Shimadzu UV-1201; Shimadzu, Kyoto, Japan). The apparent total tract digestibility (ATTD) was then calculated using the following formula: Digestibility (%) = {1 − [(Nf × Cd)/(Nd × Cf)]} × 100 Where: Nf = nutrient concentration in feces (% DM), Nd = nutrient concentration in diet (% DM), Cf = chromium concentration in feces (% DM), Cd = chromium concentration in feces (% DM).

Two pigs (one barrow and one gilt) were randomly selected from each pen and bled via jugular venipuncture at the end of the experiment (14 pigs per treatment). Blood samples (3 mL) were collected into vacuum tubes (containing no additive) (Becton Dickinson Vacutainer Systems, Franklin Lakes, NJ, USA) to obtain serum. The concentrations of triglyceride, total cholesterol, high-density lipoprotein (HDL) cholesterol, and low-density lipoprotein (LDL) cholesterol in the serum samples were analyzed with an automatic biochemical analyzer (RA-1000, Bayer Corp., Tarrytown, NY, USA) using the colorimetric method.

At the end of the experiment, fecal samples were collected directly via massaging the rectum of 2 pigs (1 gilt and 1 barrow) in each pen (14 pigs per treatment) and then pooled and placed on ice for transportation to the laboratory where analysis was immediately performed. Samples were diluted with deionized water at a ratio of 1:7.5 (w/w). One gram of the composite fecal sample from each pen was diluted with 9 mL of 1% peptone broth (Becton, Dickinson and Co., Franklin Lakes, USA) and then homogenized. Viable counts of bacteria in the fecal samples were then conducted by plating serial 10-fold dilutions (in 1% peptone solution) onto MacConkey agar plates (Difco Laboratories, Detroit, MI, USA) and Lactobacilli medium III agar plates (Medium 638; DSMZ, Braunschweig, Germany) to isolate the *E. coli* and *Lactobacillus*, respectively. The Lactobacilli medium III agar plates were then incubated for 48 h at 39 °C under anaerobic conditions. The MacConkey agar plates were incubated for 24 h at 37 °C. The *E. coli* and *Lactobacillus* colonies were counted immediately after removal from the incubator.

Fresh feces and urine samples were collected randomly from at least two pigs in each pen during the last 2 days of the end of the experiment. The urine was collected in a bucket via a funnel below the pen. Samples were kept in sealed containers and were immediately stored at −4 °C for the duration of the collection period. After the collection period, feces and urine samples were pooled and each mixed well for each pen. As described by Wang et al., subsamples of the slurry (150 g feces and 150 g of urine were mixed well; 1:1 on the wet weight basis) were taken and stored in 2.6-L plastic boxes in duplicate [19]. Each box had a small hole in the middle of one sidewall, which was sealed with adhesive plaster. The samples were permitted to ferment for 7 d at room temperature (25 °C). The concentrations of gas were determined on d 7. A gas sampling pump (Model GV-100; Gastec Corp., Ayase, Japan) was utilized for gas detection (Gastec detector tube No. 3La for ammonia (NH_3_), No. 4LK for hydrogen sulfide (H_2_S), and No. 70 for mercaptans; Gastec Corp.).

### 2.4. Statistical Analysis

The pen was used as the experiment unit and all data were subjected to statistical analysis using the GLM procedures of SAS as a randomized complete block design (SAS Institute Inc., Cary, NC, USA) [20]. Differences among all treatments were separated by Turkey’s multiple range test. A probability level of *p* < 0.05 was considered to be statistically significant.

## 3. Results 

### 3.1. Growth Performance

In the current study, pigs fed the 5:1 diet had increased (*p* < 0.05) final body weight due to greater (*p* = 0.04) ADG compared to pigs fed the 17:1 diet at the end of the experiment (Table 3). There were no significant effects (*p* > 0.05) observed in ADFI and G: F during the whole experimental period among four treatments. 

### 3.2. Nutrient Digestibility 

The digestibility of energy in the 5:1 treatment increased (*p* > 0.05) compared to pigs fed the 17:1 diet in the sixth week (Table 4). No differences (*p* > 0.05) were observed in the digestibility of DM and N among four treatments at the end of the experiment. 

### 3.3. Blood Profiles

The HDL, triglyceride and total cholesterol concentrations were not affected (*p* > 0.05) by treatments, but the LDL concentrations were decreased (*p* < 0.05) in the 5:1 treatment compared to the 17:1 and 15:1 treatment at the end of the experiment (Table 5).

### 3.4. Fecal Microflora and Fecal Noxious Gas Content

The fecal microflora (*Lactobacillus* and *E. coli*) and fecal gas emission (NH_3_, total mercaptans, and H_2_S) were unaffected (*p* > 0.0.5) by treatments at the end of the experiment (Table 6 and Table 7).

## 4. Discussions

Long-chain omega-3 PUFAs from fish oil are known to improve insulin-mediated glucose metabolism in insulin-resistant states [21,22]. The potential role in regulating insulin-mediated protein metabolism was investigated by Gingras et al., who postulated that incorporation of long-chain omega-3 PUFA into muscle membrane phospholipids of steers would enhance the sensitivity of the muscle to insulin, promote protein anabolism by reducing catabolic pathways, and enhance activation of insulin signaling [23]. Further, it is reported that in the fed steady state, chronic adaptation to long-chain omega-3 PUFA induced greater activation of the Akt-mTOR-S6K1 signaling pathway, suggesting a mechanism for increased insulin activity that induces energy utilization [9]. Finally, the efficiency of feed utilization for BW gain was increased.

The present study found that reducing omega-6:omega-3 FA ratio of growing pigs’ diets significantly increased BW due to greater ADG in the sixth week in pigs fed the 5:1 diet compared to pigs fed the 17:1 treatment because of increased energy utilization associated with insulin activity [9] and immune status improvement in this study, which is in agreement with the findings of Duan et al., who reported that the BW and ADG of pigs fed the diet with an omega-6:omega-3 PUFA ratio of 5:1 were increased significantly [11]. Upadhaya et al also showed that a lower omega-6:omega-3 PUFA ratio in the diet linearly increased BW and ADG of weanling pigs but did not affect ADFI [24]. Similarly, a significant difference for BW and ADG of piglets was observed in the fourth week in sows fed a lower omega-6: omega-3 PUFA ratio [25]. In contrast, Li et al. suggested that the inclusion of 3% omega-3 PUFA in the weaner piglet diet did not significantly improve ADG [4]. Other studies also indicated that there were no effects of dietary linseed (containing omega-3 PUFA) on ADG, feed intake or feed efficiency in pigs [26,27]. In addition, Eastwood et al indicated that there were no linear or quadratic effects of dietary flaxseed meal rich omega-3 PUFA at different levels (0, 100, 200, and 300 g of flaxseed meal/kg of diet) on basal BW gain, feed intake or feed efficiency in growing pigs [28]. The inconsistent findings regarding growth performance in pigs could be due to the oversupply of omega-6 PUFA in the diet, different feed ingredients, source of omega-3 PUFA.

The digestibility of DM and N was unaffected by reducing the omega-6: omega-3 FA ratio apart from a significant increase in energy digestibility. Similarly, Upadhaya et al. reported that reducing the omega-6: omega-3 ratio from 15:1 to 5:1 increased energy digestibility in weanling pigs [24]. In a recent study, finisher pigs fed 0.75% protected omega-3 FA also did not show improvement in the ATTD of DM and N [6]. However, our results are contrary to the findings of Upadhaya et al., who reported that the digestibility of DM and N significantly increased in weanling pigs fed the different ratios of omega-6: omega-3 PUFA diets [24]. Cho and Kim also showed that the addition of 1.5% and 3% microencapsulated omega-3 FA from linseed oil did not have any effect on the nutrient digestibility of finishing pigs [29]. The conflicting findings among studies may be related to the type of diets, age, and species of animals and the dose of omega-3 FA in the diets.

Low-density lipoprotein is a major carrier of cholesterol from the liver to each tissue. An increase of LDL seems to have an intimate relation to the generation of arteriosclerosis [30]. Therefore, the cholesterol in LDL (hereinafter referred to as ‘LDL-cholesterol’) is regarded as a risk factor for arteriosclerosis, and ischemic heart disease (coronary arteriosclerotic disease). Thus, the content of LDL-cholesterol is an important indication of diagnosis, therapy and progression of these diseases [30]. In this study, the HDL, triglyceride, and total cholesterol were unaffected in growing pigs fed the diet with different ratios of omega-6: omega-3 FA. However, the LDL concentrations of blood were significantly reduced in pigs fed the diet with omega-6: omega-3 ratio of 5:1. Similar results were reported by other researchers in human nutrition trials. For example, Pan et al., Siri-Tarino, and Avelino et al. reported that the replacement of dietary carbohydrate with PUFA from linseed oil in humans reduced levels of LDL [31,32,33]. In addition, there were no significant differences observed in HDL and triglyceride concentrations in human [34]. These results are contrary to the findings of Upadhaya et al., who reported a linear increase in HDL-cholesterol concentrations with the reduction in the ratio of omega-6: omega-3 FA in the diets but no difference was found in LDL concentrations among treatments [24]. The finding of a decrease in LDL-cholesterol concentrations with the reduction in the ratio of omega-6: omega-3 FA in the growing pigs in this study may have a beneficial effect on animal health.

The n-3 and n-6 PUFAs are well-known modulators of the inflammatory process. The PUFA have competing roles in inflammatory pathways where ahigh n-3 PUFA intake can reduce the production of proinflammatory eicosanoids derived from n-6 PUFA, partially because n-3 fatty acids are the preferential substrates for enzymes involved in eicosanoid metabolism [35]. Therefore, it is suggested that the balance of n-6 and n-3 PUFA in the diet may have stronger effects on fecal microflora than individual PUFA [25]. Yin et al. observed a significant effect on fecal *E. coli* and *Lactobacillus* counts gestation-lactating sows by altering the omega-6: omega-3 PUFA ratio in the diets [25]. An increased *Lactobacilli* content under n-3 PUFA consumption and a lower content under n-6 PUFA consumption in fish has also reported [36]. However, in this study, growing pigs fed the diets with different ratios of omega-6:omega-3 FA did not affect the *E. coli* and *Lactobacillus* counts as well as fecal gas emission, which is in agreement with the findings of Upadhaya et al., who reported that the *E. coli* and *Lactobacillus* counts were not affected by altering omega-6:omega-3 FA ratio in the diet of weaner piglets [24]. To the best of our knowledge, the reason for these findings is unknown. However, we hypothesize that the inconsistency in the findings may be due to the type of diets, age and species of animals, and the dose of omega-3 FA in the diet. 

## 5. Conclusions

In conclusion, feeding diets with lower omega-6: omega-3 FA ratios have a positive effect on the ADG, energy digestibility, and LDL concentration in plasma of growing pigs. The 5:1 omega-6: omega-3 ratio diet significantly increased BW, ADG, and energy, and decreased LDL concentrations of blood at the end of the experiment. The fecal *E. coli* and *Lactobacillus* counts, and fecal gas emission were not affected by altering the omega-6: omega-3 FA ratio in the diets of growing pigs.

## Figures and Tables

**Table 1:** Composition of basal diets (as-fed basis)

Items	Dietary Treatment (Omega-6: Omega-3 Ratio)			
	17:1	15:1	10:1	5:1
Ingredients (%)				
Corn	66.90	66.94	66.99	67.14
Soybean meal	23.69	23.66	23.65	23.62
Tallow	3.11	3.06	2.78	1.94
Molasses	3.00	3.00	3.00	3.00
Limestone	1.18	1.18	1.18	1.18
Monodicalcium phosphate	0.93	0.93	0.93	0.93
Salt	0.40	0.40	0.40	0.40
L-Met 98%	0.05	0.05	0.05	0.05
L-Lys 78%	0.32	0.32	0.32	0.32
L-Thr 98.5	0.10	0.10	0.10	0.10
L-Trp 98%	0.10	0.10	0.10	0.10
Vitamin-Mineral Premix ^a^	0.20	0.20	0.20	0.20
Choline	0.02	0.02	0.02	0.02
Omega-3 fatty acid	0.00	0.04	0.28	0.99
Calculated composition, %				
ME, kcal	3350	3350	3350	3350
Crude protein	16.00	16.00	16.00	16.00
Crude fat	5.63	5.62	5.53	5.25
Crude fiber	2.89	2.89	2.89	2.89
Crude ash	4.42	4.42	4.42	4.42
Ca	0.66	0.66	0.66	0.66
Total phosphorus	0.51	0.51	0.51	0.51
Available P	0.26	0.26	0.26	0.26
SID lysine	0.98	0.98	0.98	0.98
SID methionine	0.28	0.28	0.28	0.28
Methionine + cystine	0.56	0.51	0.51	0.51
Threonine	0.59	0.59	0.59	0.59
Tryptophan	0.17	0.17	0.17	0.17

^a^ Provided per kg of complete diet: 8000 IU vitamin A, 800 IU vitamin D3, 40 IU vitamin E, 2.0 mg vitamin K, 4.0 mg vitamin B6, 3.0 mg vitamin B12, 20 μg pantothenic acid, 15 mg niacin, and 0.02 mg biotin; 150 mg Cu 150 mg Fe, 50 mg Zn, 0.5 mg I, 0.5 mg Co, and 0.3 mg Se.

**Table 2:** Analyzed fatty acid profile of the experimental diets.

Items	Dietary Treatment (Omega-6: omega-3 Ratio)			
	17:1	15:1	10:1	5:1
C13:0, %	0.56	0.61	0.25	0.42
C14:0, %	1.40	1.32	1.08	0.78
C15:0, %	0.29	0.27	0.24	0.00
C16:0, %	20.84	21.08	21.07	22.03
C18:0, %	8.48	8.91	10.83	11.83
C18:1, %	37.67	36.74	35.72	31.86
Omega-3 (linoleic acid), %	28.74	28.53	27.80	26.59
Omega-6 (α-linolenic acid), %	1.67	1.84	2.77	5.32
Docosahexaenoic acid, %	0.00	0.00	0.00	0.41
Saturated fatty acid, %	31.57	32.19	33.46	35.06
Monounsaturated fatty acid, %	37.67	36.74	35.72	31.86
Polyunsaturated fatty acid, %	30.41	30.37	30.57	32.32
Omega-6: omega-3 ratio, %	17.21	15.51	10.04	4.64

**Table 3:** Effect of omega-6: omega-3 fatty acid ratio on growth performance in growing pigs

Items	Dietary Omega-6: omega-3 Fatty Acid Ratio				SEM	*p-*Value
	17:01	15:1	10:1	5:1		
Body weight, kg						
Initial	24.75	24.76	24.74	24.74	0.01	0.17
Day 42	54.27 ^b^	54.83 ^ab^	55.29 ^ab^	55.83 ^a^	0.36	0.04
Overall						
ADG, g	703 ^b^	716 ^ab^	727 ^ab^	740 ^a^	8.70	0.04
ADFI, g	1626	1637	1661	1656	19.37	0.56
G: F	0.433	0.437	0.438	0.447	0.01	0.28

^1^ Dietary treatments were different ratio of omega-6:omega-3 fatty acid, including 17:1, 15:1, 10:1, and 5:1, SEM: standard error of means, ADG: average daily gain, ADFI: average daily feed intake, G:F: gain to feed ratio, ^a,b^ means in the same row with different superscript differ significantly (*p* < 0.05).

**Table 4:** Effect of omega-6: omega-3 fatty acid ratio on nutrient digestibility in growing pigs

Items, %	Dietary Omega-6: omega-3 Fatty Acid Ratio				SEM	*p*-Value
	17:01	15:1	10:1	5:1		
Day 42						
Dry matter	77.26	77.07	78.49	77.88	0.74	0.89
Nitrogen	76.02	75.53	77.72	78.19	0.86	0.70
Energy	76.93 ^b^	76.88 ^b^	78.48 ^ab^	78.64 ^a^	0.46	0.03

Dietary treatments were different ratio of omega-6: omega-3 fatty acid, including 17:1, 15:1, 10:1, and 5:1, SEM: standard error of means, ^a,b^ means in the same row with different superscript differ significantly (*p* < 0.05).

**Table 5:** Effect of omega-6: omega-3 fatty acid ratio on blood profile in growing pigs

Items, mg/dL	Dietary Omega-6: Omega-3 Fatty Acid Ratio				SEM	*p* -Value
	17:01	15:1	10:1	5:1		
Day 42						
HDL	38	41	44	45	3.24	0.46
LDL	59 ^a^	54 ^a^	52 ^ab^	46 ^b^	1.98	0.01
Triglyceride	46	45	42	40	3.27	0.50
Total cholesterol	119	114	117	118	4.89	0.88

Dietary treatments were different ratio of omega-6: omega-3 fatty acid, including 17:1, 15:1, 10:1, and 5:1, SEM: standard error of means, HDL: high-density lipoprotein, LDL: low-density lipoprotein, ^a,b^ means in the same row with different superscript differ significantly (*p* < 0.05).

**Table 6:** Effect of omega-6: omega-3 fatty acid ratio on fecal microbial population in growing pigs

Items, log_10_cfu/g	Dietary Omega-6: Omega-3 Fatty Acid Ratio				SEM	*p*-Value
	17:01	15:1	10:1	5:1		
Day 42						
*Lactobacillus*	7.37	7.41	7.45	7.50	0.05	0.39
*E. coli*	5.90	5.89	5.89	5.86	0.07	0.97

Dietary treatments were different ratio of omega-6: omega-3 fatty acid, including 17:1, 15:1, 10:1, and 5:1, SEM: standard error of means.

**Table 7:** Effect of omega-6: omega-3 fatty acid ratio on fecal gas-emission in growing pigs

Items, ppm	Dietary Omega-6: omega-3 Fatty Acid Ratio				SEM	*p*-Value
	17:01	15:1	10:1	5:1		
Week 6						
NH_3_	4.75	4.73	4.40	4.33	0.66	0.95
Total mercaptans	3.15	2.90	2.80	2.85	0.43	0.94
H_2_S	3.50	3.18	3.33	3.20	0.70	0.99

Dietary treatments were different ratio of omega-6: omega-3 fatty acid, including 17:1, 15:1, 10:1, and 5:1, SEM: standard error of means, NH_3_: ammonia, H_2_S: hydrogen sulfide.
